# Critical role of Rho proteins in myosin light chain di-phosphorylation during early phase of endothelial barrier disruption

**DOI:** 10.1186/s12576-022-00857-x

**Published:** 2022-12-07

**Authors:** Mayumi Hirano, Katsuya Hirano

**Affiliations:** Department of Cardiovascular Physiology, Faculty of Medicine, Kagawa University, Miki-cho, Kita-Gun, Kagawa Japan

**Keywords:** Vascular endothelial cells, Barrier function, Myosin light chain, Phosphorylation, Small G protein, Actin filaments

## Abstract

We previously reported the Rho-associated coiled-coil containing protein kinase (ROCK)-mediated di-phosphorylation of myosin light chain (MLC) and actin bundle formation at the cell periphery as early events of the endothelial barrier disruption. We herein examined the role of RhoA during early events of barrier disruption. Treatment of cultured porcine aortic endothelial cells with simvastatin prevented the decrease in trans-endothelial electrical resistance, MLC di-phosphorylation and peripheral actin bundle formation seen 3 min after thrombin stimulation. Co-treatment with geranylgeranyl pyrophosphate rescued the thrombin-induced events. Thrombin increased a GTP-bound form of RhoA and phosphorylation of myosin phosphatase target subunit 1 (MYPT1) at the ROCK site. The intracellular introduction of the inhibitory protein of RhoA inhibited the thrombin-induced di-phosphorylation of MLC. However, knockdown of either one of RhoA, RhoB or RhoC failed to inhibit thrombin-induced MLC di-phosphorylation. The findings suggest that Rho proteins play a critical role during early events of thrombin-induced barrier disruption.

**Supplementary Information:**

The online version contains supplementary material available at 10.1186/s12576-022-00857-x.

## Background

Vascular endothelial cells form a monolayer sheet lining the luminal surface of the blood vessels, and play a critical role in the regulation of vascular permeability [1–5]. Under physiological conditions, quiescent endothelial cells maintain the regulated permeability of the paracellular pathway by forming a tight cell-to-cell junction, with prominent circumferential actin filaments that run in parallel to the junction, but with a low level of phosphorylation of 20 kDa myosin light chain (MLC) and scarce actin stress fibers that run through the cells [5]. This quiescent situation is associated with an increased activity of Rac1 and a decreased activity of RhoA [5]. Under pathological conditions, disruption of the paracellular pathway plays a major role in increasing the vascular permeability. The loss of the integrity of the cell-to-cell junction, downregulation of circumferential actin filaments, increase in MLC phosphorylation, formation of actin stress fibers, decrease in Rac1 and increase in RhoA activities cooperatively contribute to the disruption of the paracellular pathway and an increase in vascular permeability. The formation of actin stress fibers and actomyosin force generation due to MLC phosphorylation induce the contraction of endothelial cells, thereby causing intercellular gap formation [1–5]. The phosphorylation of MLC at either S19 or T18 increases the myosin ATPase activity, thereby causing actomyosin force generation [6]. Phosphorylation at S19 plays a primary role, while phosphorylation at T18 plays an additive role. MLC is mono-phosphorylated at S19 or di-phosphorylated at S19 and T18; no mono-phosphorylation at T18 has never been observed in the cellular context [6, 7].

In the previous study, we identified previously unappreciated early events during thrombin-induced barrier disruption [7]. Namely, we showed that thrombin specifically induced MLC di-, but not mono-phosphorylation and actin bundle formation at the cell periphery before inducing actin stress fiber formation in the late phase. In that study, we proposed that the MLC di-phosphorylation and actin bundle formation at the cell periphery caused a concentric force generation during the early phase, which subsequently loosened the cell–cell contact, thereby reorganizing peripheral actin bundles to stress fibers in the later phase. However, the mechanism that restricted MLC di-phosphorylation at the cell periphery remained unclear.

The protein kinases that are capable of inducing MLC di-phosphorylation include Ca^2+^-calmodulin-dependent MLC kinase (MLCK), Rho-associated coiled-coil containing protein kinase (ROCK), zipper-interacting protein kinase (ZIPK) and integrin-linked protein kinase (ILK) [8–11]. Our previous study proposed an essential role of ROCK in MLC di-phosphorylation and actin bundle formation at the cell periphery, since the ROCK inhibitors Y27632 and H1152 abolished these events [7]. However, the role of ROCK in the formation of stress fibers has been well established [12, 13]. ROCK can localize at the cell membrane, as ROCK is activated by interaction with RhoA as well as lipids, such as arachidonic acid [13, 14]. The GTP-bound active form of RhoA localizes at the plasma membrane via a geranylgeranyl moiety. The membrane localization of ROCK is thus due in part to the membrane localization of active RhoA and in part to interaction with lipids via its pleckstrin-homology (PH) domain [15]. As a result, it is hypothesized that the localization of RhoA plays a primary role in restricting ROCK and thereby MLC di-phosphorylation at the cell periphery during the early events of thrombin-induced barrier disruption.

The present study addressed the critical involvement of RhoA in the early events of the thrombin-induced endothelial barrier disruption. In order to inhibit membrane localization of RhoA, cells were pretreated with simvastatin, an inhibitor of 3-hydroxy-3-methyl-glutaryl CoA reductase that not only inhibits de novo synthesis of cholesterol, but also depletes farnesyl pyrophosphate (FPP), an intermediate metabolite of cholesterol synthesis, thereby depleting geranylgeranyl pyrophosphate (GGPP) [16]. The inhibitory protein of RhoA was also used to specifically evaluate the involvement of RhoA. In order to examine the specific involvement of RhoA, the effect of knockdown of RhoA, RhoB and RhoC on MLC di-phosphorylation was examined.

## Methods

### Materials

**Fig. 1:**
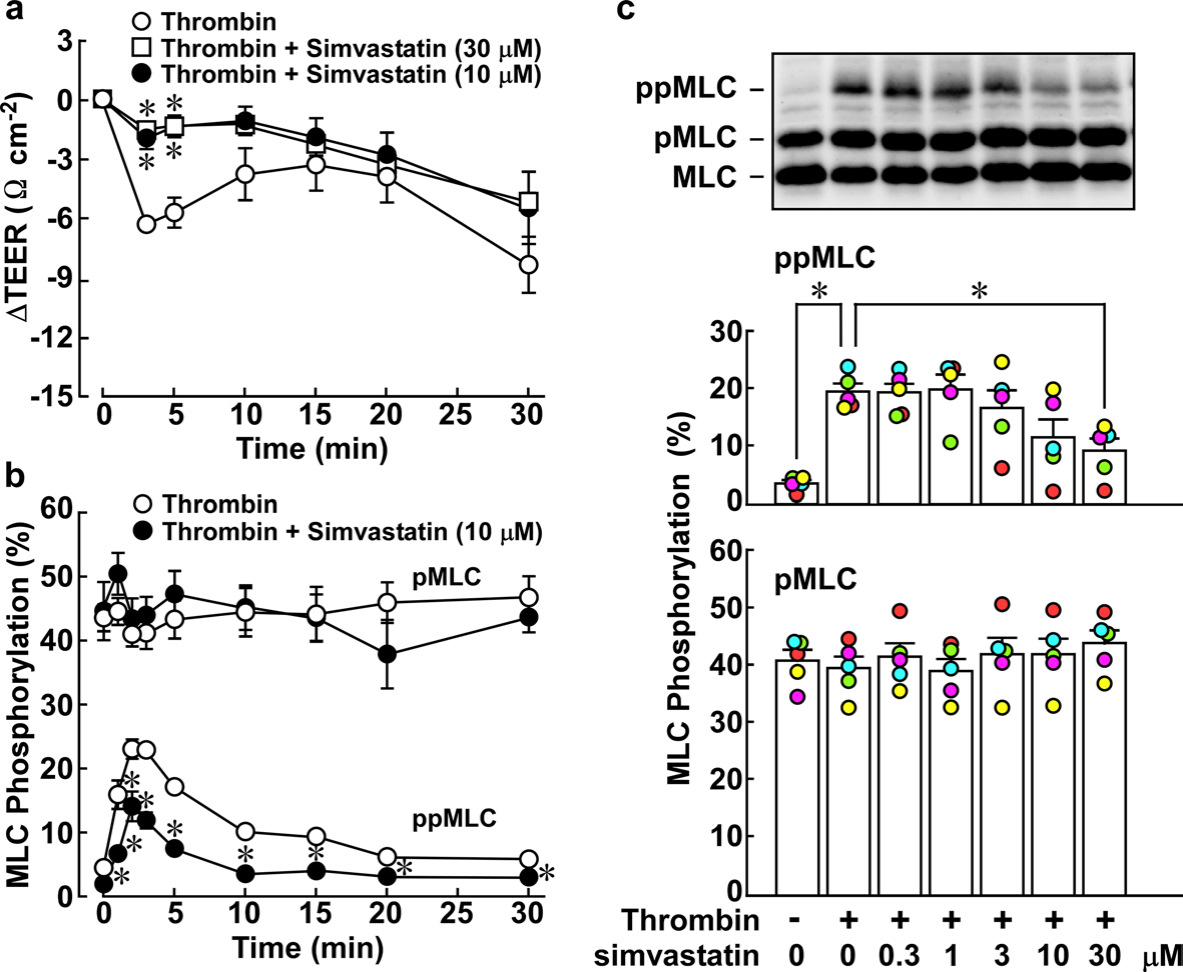
Effects of simvastatin pretreatment on the thrombin-induced decrease in trans-endothelial electric resistance (TEER) and mono- and di-phosphorylation of myosin light chain (pMLC, ppMLC) in porcine aortic endothelial cells (PAECs). **a**, **b** The time courses of thrombin-induced changes in TEER (a, *n* = 6), pMLC and ppMLC (b, *n* = 4) are summarized. **c** A representative immunoblot and the summary for the concentration-dependent effects of simvastatin on pMLC and ppMLC at 3 min after thrombin stimulation are shown (*n* = 5). PAECs at confluence were pretreated or left untreated with simvastatin at the indicated concentrations for 16 h. The cells were then stimulated with 1 unit/mL thrombin, in the presence of simvastatin, when pretreated. All original images of the immunoblot analyses are shown in Additional file 1: Figure S1. The data are expressed as the mean ± SEM. *P < 0.05 vs. thrombin, according to an ANOVA followed by Dunnett’s post hoc test

**Fig. 2:**
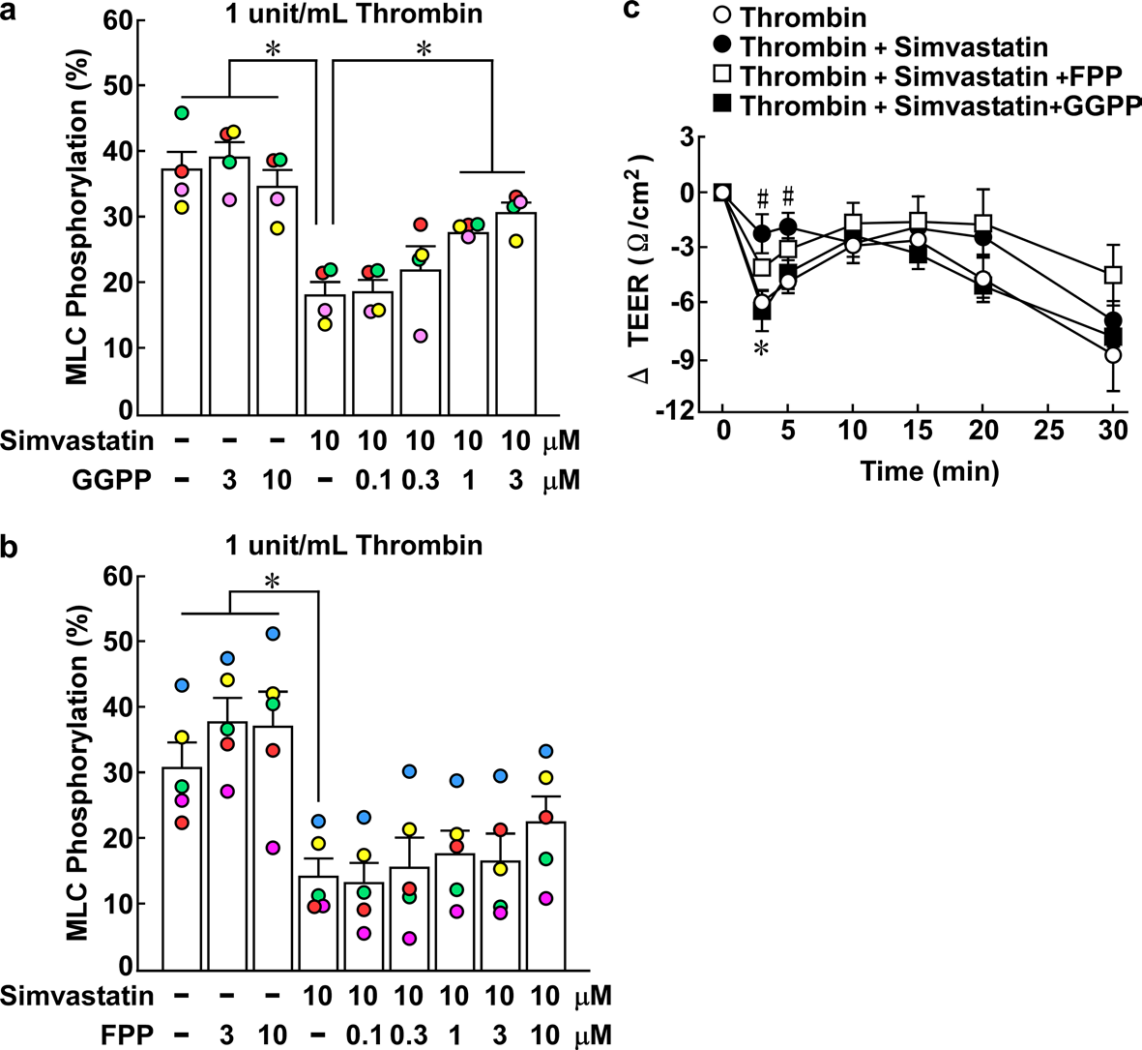
Effects of geranylgeranyl pyrophosphate (GGPP) and farnesyl pyrophosphate (FPP) on the inhibition by simvastatin of the thrombin-induced di-phosphorylation of myosin light chain (ppMLC) and decrease in the trans-endothelial electrical resistance (TEER) in porcine aortic endothelial cells (PAECs). **a**, **b** The effects of simvastatin and GGPP (a; *n* = 4) or FPP (b; *n* = 5) on the thrombin-induced ppMLC were summarized. PAECs were pretreated or left untreated with simvastatin, GGPP or FPP, either alone or their combination, at the indicated concentrations for 16 h. The cells were then stimulated with 1 unit/mL thrombin, in the presence of simvastatin, GGPP or FPP, when pretreated. The levels of ppMLC were evaluated 3 min after thrombin stimulation. All original images of the immunoblot analyses in panels a and b are shown in Additional file 1: Figure S2. **c** Shown are the time courses of the change in the TEER after stimulation with 1 unit/mL thrombin in PAECs with and without pretreatment with 10 µM simvastatin, 3 µM GGPP or 10 µM FPP, as indicated (*n* = 5). The data are expressed as the mean ± SEM. *P < .05 vs. simvastatin (**a**, **b**) and thrombin + simvastatin (**c**); #*P* < 0.05 vs. thrombin (**c**), according to an ANOVA followed by Dunnett’s post hoc test

Thrombin (T7513, bovine plasma), simvastatin (S6196), GGPP ammonium salt (G6025) and FPP (F6892) were purchased from Sigma (St. Louis, MO, USA). Y27632 and H1152 glycyl were purchased from Merck Millipore (Billerica, MA, USA). The stock solutions of reagents were as follows: 10 mM simvastatin in H_2_O, 2.2 mM GGPP in methanol, 2.3 mM FPP in methanol, 1 unit/µl thrombin in H_2_O, 10 mM Y27632 and 1 mM H1152 in H_2_O. When the concentration-dependent effects of simvastatin (Fig. 1c), and GGPP and FPP (Fig. 2a, b) were examined, serial dilutions were prepared in the solvent, and the same volume of such serial dilutions was applied to the cells. The primary antibodies used were an anti-pan MLC antibody (sc-15370; Santa Cruz, Dallas, TX, USA), anti-phospho-MLC antibody at T18 and S19 (#3674; Cell Signaling, Beverly, MA, USA), mouse monoclonal anti-VE-cadherin antibody (sc-9989, Santa Cruz), anti-RhoA antibody (#ARH04 Cytoskeleton, Denver, CO. USA), anti-MYPT1 antibody (#612164; BD Biosciences, Franklin Lakes, NJ, USA), and anti-phospho-MYPT1(Thr850) antibody (#36-003; Millipore, Temecula, CA, USA). The secondary antibodies conjugated with horseradish peroxidase were purchased from Sigma. Anti-mouse IgG antibodies conjugated with Alexa488 (#4408) or Alexa647 (#4410) and anti-rabbit IgG antibodies conjugated with Alexa488 (#4412) or Alexa647 (#4414) were purchased from Cell Signaling Technology (Danvers, MA, USA). Anti-mouse IgG and anti-rabbit IgG antibodies conjugated with Alexa 546 (A11018 and A11071, respectively), FITC-phalloidin was purchased from Life Technologies-Molecular Probes Japan (Tokyo, Japan).

### Cell culture and experimental protocol

Porcine aortas were obtained at a local slaughterhouse, and porcine aortic endothelial cells (PAECs) were established and cultured in Dulbecco’s modified Eagle’s medium (Sigma) supplemented with 10% fetal bovine serum, as previously described [7, 17]. The cells obtained at passages 9–16 from the original isolation of the cells from the tissues were used in all investigations, as previously described [7, 17]. Cells were plated at the density indicated in the following sections and cultured for 6–8 days until confluent. The cells were then serum-starved overnight before the day of the experiment. The 16-h pretreatment with simvastatin, GGPP and FPP was performed during this serum-free incubation. Cells were challenged with thrombin in the presence of simvastatin, GGPP or FPP, when pretreated. Human microvascular endothelial cells (HMVECs) were purchased from Kurabo (KE-4209; Osaka, Japan) and cultured in HuMedia-MvG media (KE-2550; Kurabo). The cells at passage 4 and 5 were used in the experiments.

### Knockdown of RhoA, RhoB and RhoC by small interfering RNA (siRNA)

siRNAs targeting porcine RhoA (Accession No. NM_001244437.1), RhoB (NM_001123189.1) and RhoC (XM_003125846.4) were designed and synthesized by Sigma-Aldrich Japan (Tokyo, Japan). The sequences of sense siRNAs are AUA CUG AUG UUA UAC UGA U (RhoA), CCG ACG UGA UCC UUA UGU G (RhoB) and AUC CUC AUG UGC UUC UCC A (RhoC), all with overhang of two deoxythymidines. siRNA Universal Negative Control #1 (Sigma-Aldrich Japan) was used for negative control experiments. PAECs were plated at 3 × 10^5^ cells/60 mm-dish, the medium was replaced on the next day and the cells were exposure to 10 nM siRNA for 3 days. Cells were harvested and homogenized before and after thrombin stimulation as described below in the Preparation of cell lysates for immunoblot analysis section and MLC di-phosphorylation was analyzed with immunoblot.

### Measurement of the trans-endothelial electrical resistance (TEER)

The endothelial barrier function was evaluated by assessing TEER, as previously described [7]. In brief, PAECs were plated at 1.5 × 10^4^ cells in 0.32-cm^2^ culture inserts with polyethylene terephthalate membranes (0.4 µm pore size, 1.6 × 10^8^ pores cm^−2^) in 24-well culture plates (BD Falcon, Tokyo, Japan). TEER measurements were conducted in Hepes-buffered saline (HBS: 10 mM Hepes at pH 7.4, 135 mM NaCl, 5 mM KCl, 1 mM CaCl_2_, 1 mM MgCl_2_, 5.5 mM D-glucose) at 37 °C with an EVOM2 voltohmmeter equipped with a STX2 electrode (World Precision Instruments, Sarasota, FL USA). The cells were equilibrated in HBS for 10 min prior to the experimental protocol. The change in the TEER (ΔTEER) from the value obtained in HBS just prior to agonist stimulation was expressed as the absolute value of the resistance normalized by the surface area of the culture insert membrane (Ω cm^−2^), unless otherwise specified.

### Preparation of cell lysates for immunoblot analysis

The cells were plated at 3 × 10^5^ cells in 60-mm culture dishes. Cells were rinsed three times with pre-warmed phosphate-buffered saline (PBS; 136.9 mM NaCl, 2.7 mM KCl, 8.1 mM Na_2_HPO_4_, 1.47 mM KH_2_PO_4_), and then equilibrated for further 10 min and subjected to the experimental protocols in HBS at 37 °C. At each time point, the cells were rinsed once with ice-cold PBS containing 0.1 mM EDTA to remove residual extracellular Ca^2+^. The cells were then lysed in 70 µL ice-cold lysis buffer containing 50 mM Hepes, 150 mM NaCl, 0.5% Nonidet P-40, 1 mM dithiothreitol, 10 µg/ml leupeptin, 10 µg/ml aprotinin, 10 µM 4-amidinophenylmethanesulfonyl fluoride (p-APMSF), 20 µM NaF, 50 µM calpain inhibitor I and 5 µM microcystin-LR. The lysates were immediately collected using a cell scraper, transferred to a microcentrifuge tube and snap-frozen in liquid N_2_. The lysates were then thawed on ice for 20 min and centrifuged at 12,000 rpm for 20 min on microcentrifuge (Type 1120; Kubota, Tokyo, Japan) in a cold room. The supernatants were then made the samples for sodium dodecyl sulfate-polyacrylamide gel electrophoresis (SDS-PAGE). The protein concentrations of supernatants were estimated with a Coomassie (Bradford) protein assay kit (Thermo Fisher Scientific Pierce, Rockford, IL, USA).

### Phos-tag SDS-PAGE and the immunoblot analysis of MLC phosphorylation

The mono- and di-phosphorylation of MLC (pMLC and ppMLC, respectively) were quantitatively analyzed using Phos-tag SDS-PAGE followed by immunoblot detection, as previously reported [7]. In brief, the cell lysate (10 µg protein) was subjected to Phos-tag SDS-PAGE on 10% polyacrylamide gels containing 30 µM Phos-tag and 60 µM MnCl_2_. After electrophoresis, the gels were soaked for 30 min at room temperature in transfer buffer (25 mM Tris-hydroxymethyl aminomethane, 192 mM glycine, 20% methanol) containing 2 mM EDTA to remove Mn^2+^ and then soaked in the transfer buffer for 15 min. Proteins were then transferred to polyvinylidene difluoride membranes (Merck Millipore). The membranes were post-fixed in PBS containing 0.5% formaldehyde for 45 min [7], rinsed once with PBS containing 0.1% Tween-20 (T-PBS), and then blocked with 5% non-fat dry milk in T-PBS overnight in a cold room. The membranes were incubated with anti-pan MLC antibody (x 2000) and then secondary antibodies (x 2000) for 60–90 min in each step at room temperature. The immune complexes were detected with an ECL Plus or ECL Select detection kit (GE Healthcare, Buckinghamshire, UK). The chemiluminescence emission was captured with a ChemiDoc XRS-J instrument and analyzed using the Quantity One software program (Bio Rad, Hercules, CA, USA). The percentages of pMLC and ppMLC in the total MLC (a sum of the optical densities of non-phosphorylated MLC, pMLC and ppMLC, all of which were detected with the anti-pan MLC antibody) were calculated for each lane.

### Immunoblot analysis of MLC di-phosphorylation

The thrombin-induced di-phosphorylation of MLC in PAECs, which were subjected to knockdown of RhoA, RhoB and RhoC, was analyzed using anti-phospho-MLC at T18 and S19 antibody. The conventional SDS-PAGE with 3 μg protein loading and protein transfer to polyvinylidene difluoride membranes (Merck Millipore; Billerica, MA, USA) were performed as previously described [7]. Total level of MLC was analyzed with anti-pan MLC antibody. Since the levels of ppMLC and total amount of MLC were analyzed on separate membranes, tubulin was detected as an internal control for normalization of the corresponding levels of ppMLC and total MLC, respectively. The chemiluminescence signal was captured and analyzed as described above. The ratio of the tubulin-normalized level of ppMLC and that of total MLC was expressed as a relative value, while assigning the values obtained with the negative control siRNA to be 1.

### The immunoblot analysis of the phosphorylation of MYPT1 at the site corresponding to T853 of human MYPT1

The cell lysates (10 µg protein) were subjected to conventional SDS-PAGE on 10% polyacrylamide gels and then transferred to polyvinylidene difluoride membranes, as described above. After fixation with 0.5% formaldehyde in PBS for 45 min and rinsing in T-PBS, the membranes were blocked with Tris-buffered saline (TBS; 20 mM Tri–HCl, pH7.5, 150 mM NaCl) containing 0.05% Tween 20 (T-TBS) supplemented with 5% bovine serum albumin for the detection of total MYPT1 or T-TBS supplemented with 5% non-fat dry milk for the detection of phosphorylated from of MYPT1. The membranes were then subjected to an immunoblot analysis using anti-MYPT1 antibody (x 5000 dilution) and anti-phospho-MYPT1 (Thr850) antibody (x 1000 dilution). The immune complexes were detected and analyzed, as described above. The level of MYTP1 phosphorylation was normalized by the level of total MYPT1, and then expressed as a relative value, while assigning the basal level obtained before thrombin stimulation a value of 1.

### A pull-down assay of the GTP-bound form of RhoA

The level of GTP-bound form of RhoA was quantified with a pull-down assay using a Rho Activation Assay Biochem Kit (Cytoskeleton, Denver, CO, USA), according to the manufacturer’s instructions. The level of the GTP-bound form of RhoA was normalized by the level of total RhoA, and then the basal level of the GTP-bound form of RhoA was assigned a value of 1.

### Immunofluorescence staining

PAECs and HMVECs were plated at 1.5 × 10^5^ cells on glass coverslips (22 × 22 mm, Matsunami, Osaka, Japan) in 35 mm culture dishes. Fluorescence staining of phosphorylated MLC, VE-cadherin and F-actin was performed, as previously reported [7]. 4′,6-diamidino-2-phenylindole (DAPI), included in ProLong Gold antifade mount (Thermo Fisher Scientific), or Topro-3 (Thermo Fisher Scientific) was used to stain nucleus as indicated. The confocal fluorescence images of PAECs and HMVECs were captured with a Nikon confocal laser microscope A1 with a 60x objective lens (Tokyo, Japan) and Carl Zeiss LSM 700 with a 40x objective lens (Oberkochen, Germany), respectively. The original images of PAECs were saved in the JPEG2000 format using the imaging software program NIS-elements AR (Nikon). The images used for presentation were exported from the original files and saved in the TIFF format using the Snapshot function of NIS-elements AR. The original images of HMVECs were obtained with the imaging software program ZEN (Carl Zeiss) and exported as TIFF files for presentation. The images of actin and ppMLC in PAECs, both of which were in a green channel, were extracted by setting the intensities of red and blue channels to 0 and subjected to quantification of the intensity of fluorescence with an Image J ver.1.53 k software (National Institute of Health, Bethesda, MD, USA). The values were expressed as a percentage of the control value.

### Preparation of inhibitory proteins of RhoA and Rac1/Cdc42 as fusion proteins with the cell-penetrating peptide derived from human immunedeficiency viral Tat protein

The RhoA-binding domain of ROCK (amino acid residues 941-1075 in humans) and the Rac1/Cdc42-binding domain of p21-activated protein kinase 1 (amino acid residues 67-150 in humans) were prepared as fusion proteins with a (His)_6_ tag, cell-penetrating peptide of human immunodeficiency viral Tat protein and a hemagglutinin tag at their N-terminus for the inhibitory proteins of RhoA and Rac1/Cdc42, respectively, as previously described [18–21]. The proteins were expressed in *E. coli* strain BL21(DE3)LysS and affinity-purified with Ni^2+^-loaded Hi-Trap chelating column on Akta Prime (Cytiva, Tokyo, Japan). The column eluate (20 mM Tris–HCl, 500 mM imidazole, 500 mM NaCl, 1 mM 2-mercaptoethanol, pH 8.0) of the inhibitory protein of Rac1/Cdc42 was directly used in the experiment, while the column eluate of the inhibitory protein of RhoA was dialyzed against PBS and the dialysate was used in the experiment. The protein concentrations of the inhibitory proteins of RhoA and Rac1/Cdc42 were determined to be 186 μM and 475 μM, respectively, with the Bradford method using bovine serum albumin as the standard (Pierce, Rockford, IL, USA). PAECs were exposed to the protein preparations in HBS for 30 min prior to and during stimulation with 1 unit/mL thrombin.

### Statistical analyses

The data are presented as means ± standard error of the mean (SEM) of the indicated number of independent experiments. In bar graphs, the circles overlaid on columns indicate individual data points, while data points in the same color are derived from the same set of experiments. The StatView ver.5 software program (SAS Institute, Cary, NC, USA) was used to evaluate the statistical significance by an analysis of variance (ANOVA), followed by the Dunnett's post hoc test for comparisons to control condition and the Fisher post hoc test for multiple comparisons, as indicated in the figure legends. A value of *P* < 0.05 was considered to be statistically significant.

## Results

### Simvastatin treatment prevented the thrombin-induced decrease in trans-endothelial electrical resistance (TEER) and MLC di-phosphorylation in PAECs

PAECs were treated with simvastatin for 16 h, and then stimulated with 1 unit/mL thrombin in the presence of simvastatin. The concentration of thrombin used in the present study is the optimal concentration to induce the maximal effects on TEER and MLC phosphorylation, as previously reported [7]. Simvastatin treatment substantially inhibited the thrombin-induced decrease in TEER, especially in the early phase (3–10 min after thrombin stimulation) (Fig. 1a). A concentration of 10 µM was sufficient to exert the maximal inhibition. This concentration of simvastatin significantly inhibited the thrombin-induced di-phosphorylation of MLC but had no effect on mono-phosphorylation (Fig. 1b). Simvastatin exerted concentration-dependent inhibition of MLC di-phosphorylation, reaching significant inhibition at 30 µM (Fig. 1c).

### GGPP prevented simvastatin-caused inhibition of MLC di-phosphorylation and decrease in TEER induced by thrombin in PAECs

To obtain some mechanistic insight into the inhibitory effect of simvastatin on the thrombin-induced MLC phosphorylation and decrease in TEER, the antagonizing effects of GGPP and FPP were investigated. MLC di-phosphorylation induced by 1 unit/mL thrombin was significantly decreased in PAECs pretreated with 10 µM simvastatin for 16 h (Fig. 2a, b). When PAECs were co-treated with simvastatin and GGPP, the inhibitory effect of simvastatin was prevented in a manner dependent on the concentration of GGPP (Fig. 2a). In contrast, FPP had no effect on the inhibitory effect of simvastatin in thrombin-induced MLC di-phosphorylation, even at 10 µM (Fig. 2b).

The preventive effects of GGPP and FPP were also examined regarding the thrombin-induced decrease in TEER (Fig. 2c). The inhibition of the thrombin-induced decrease in TEER by 10 µM simvastatin was abolished by co-treatment with 3 µM GGPP, while 10 µM FPP exerted no significant preventive effect (Fig. 2c).

### Simvastatin inhibited thrombin-induced MLC di-phosphorylation and actin bundle formation at the cell periphery in a manner sensitive to GGPP in PAECs

**Fig. 3:**
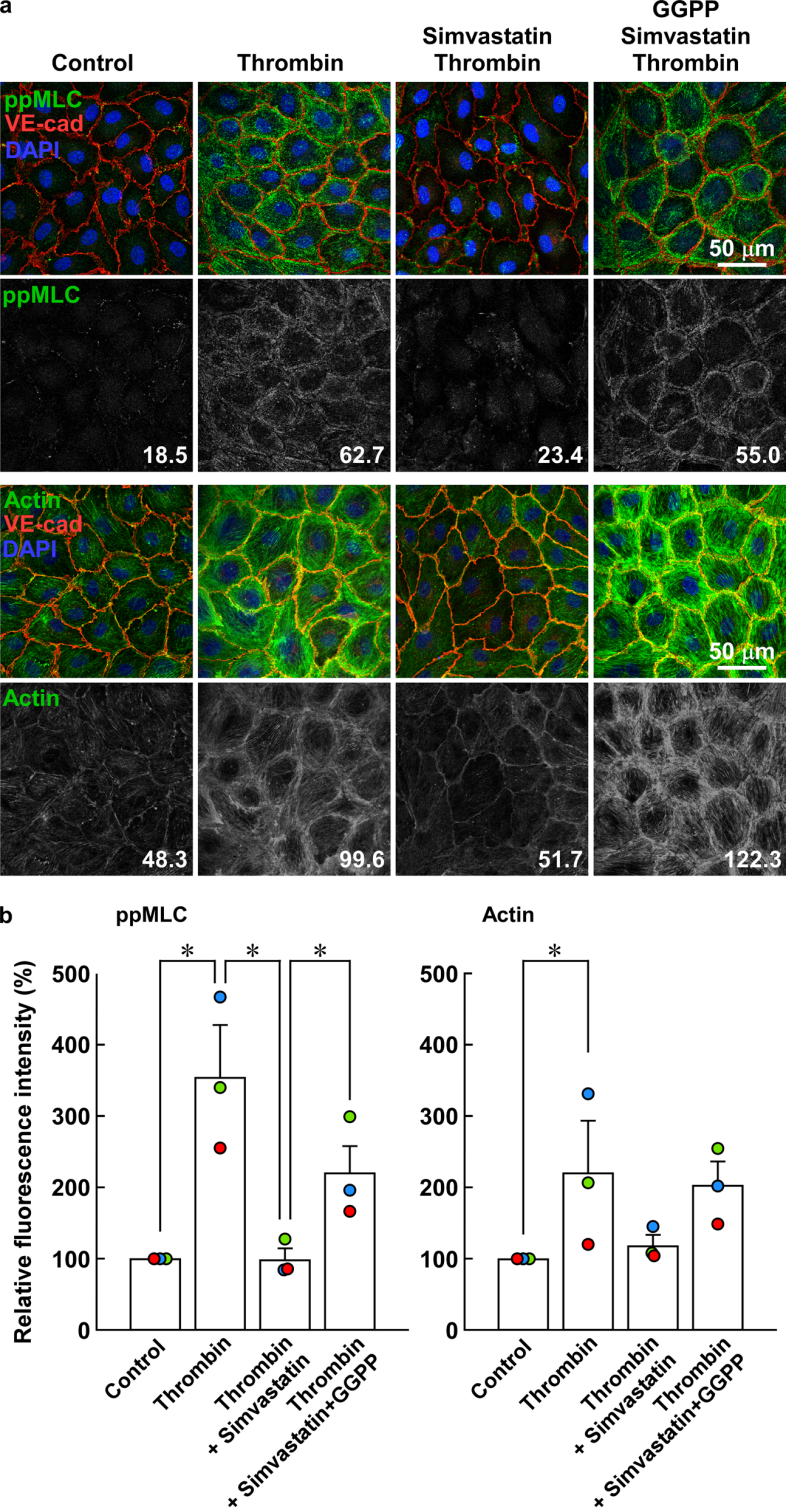
Effects of simvastatin and geranylgeranyl pyrophosphate (GGPP) on localization of di-phosphorylation of myosin light chain (ppMLC) and actin bundle formation in porcine aortic endothelial cells (PAECs). **a** Representative merged images of confocal microphotographs show the triple fluorescence staining of either F-actin/VE-cadherin (VE-Cad)/nuclei (DAPI) or ppMLC/VE-Cad/DAPI in PAECs in the indicated colors. The extracted images of ppMLC and actin (both in a green channel) in a gray scale are shown below the corresponding merged images. The readings of the fluorescence intensity were indicated at a lower right corner of each image. **b** Summaries (*n* = 3) of quantitative analysis of fluorescence intensities of ppMLC and actin. The data are expressed as the mean ± SEM. *P < 0.05 according to an ANOVA followed by Fisher post hoc test. PAECs were either pretreated or left untreated with 10 µM simvastatin for 16 h with and without co-treatment with 3 µM GGPP (*n* = 3). The images were obtained 3 min after stimulation with 1 unit/mL thrombin. The cells were challenged to thrombin, in the presence of simvastatin and GGPP, when pretreated. Control indicates images of cells without any treatment. Thrombin induced peripheral accumulation of ppMLC and actin bundles in close proximity to VE-cadherin. Simvastatin inhibited the thrombin-induced peripheral accumulation of ppMLC and actin bundles, while co-treatment with GGPP prevented the simvastatin effects. The results obtained in the two additional sets of the experiments are shown in Additional file 1: Figure S3

Thrombin caused MLC di-phosphorylation and actin bundle formation at the cell periphery at 3 min after stimulation, both of which were absent before thrombin stimulation (Fig. 3a). Pretreatment with 10 µM simvastatin abolished the localization of MLC di-phosphorylation and actin bundle formation at the cell periphery after thrombin stimulation, while 3 µM GGPP prevented the inhibitory effect of simvastatin (Fig. 3a). These changes in ppMLC and actin were supported by quantitative analysis of fluorescence intensities (Fig. 3b).

Microvascular endothelial cells represent the major site regulating vascular permeability in vivo, especially under inflammation. The peripheral actin bundle formation was also observed as an early event after thrombin stimulation in HMVECs as in the case of PAECs (Additional file 1: Figure S7).

### Activation of the RhoA–ROCK pathway during the early phase of thrombin-induced barrier disruption in PAECs

**Fig. 4:**
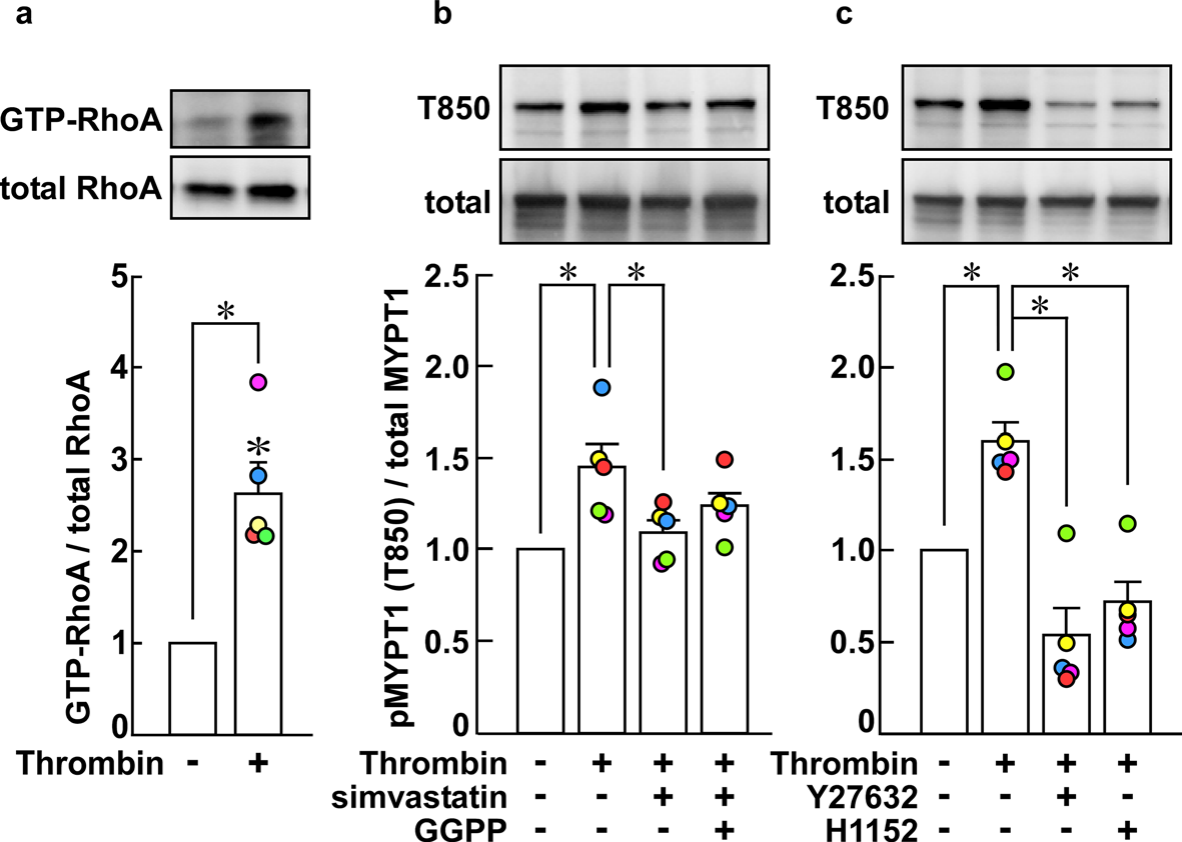
Activation of RhoA and Rho-associated coiled-coil containing protein kinase (ROCK) by thrombin in porcine aortic endothelial cells (PAECs). **a** The levels of GTP-bound form of RhoA before and 3 min after stimulation with 1 unit/mL thrombin were summarized (*n* = 5). **b**, **c** Shown are the representative immunoblots and the summaries of the level of phosphorylation of MYPT1 at the ROCK site corresponding to T850 in human MYPT1 before and 3 min after stimulation with 1 unit/mL thrombin in the presence and absence of 10 µM simvastatin and 3 µM GGPP (**b**; *n* = 5), and 10 µM Y27632 and 1 µM H1152 (**c**; *n* = 5). PAECs were pretreated with simvastatin for 16 h in the presence or absence of GGPP, and with Y27632 or H1152 for 30 min, and then stimulated with thrombin in their presence, when pretreated (*n* = 5). The basal level of MYPT1 phosphorylation was assigned a value of 1. The data are expressed as the mean ± SEM. *P < 0.05 vs. thrombin, according to an ANOVA followed by Dunnett’s post hoc test. All original images of the immunoblot analyses are shown in Additional file 1: Figure S4

Thrombin increased the level of the GTP-bound form of RhoA in PAECs 3 min after stimulation (Figs. 4a). The thrombin activation of ROCK was supported by an increase in the phosphorylation of MYPT1 at the ROCK site (T850 in human MYPT1) (Fig. 4b). The thrombin-induced MPYT1 phosphorylation was inhibited by pretreatment with 10 µM simvastatin, while co-treatment with 3 µM GGPP did not significantly prevent this inhibition (Fig. 4b). The involvement of ROCK in the thrombin-induced MYPT1 phosphorylation was validated by the inhibitory effect of two inhibitors of ROCK: Y27632 and H1152 (Fig. 4c).

### Introduction of inhibitory protein of RhoA, but not Rac1/Cdc42, inhibited thrombin-induced di-phosphorylation of MLC in PAECs

**Fig. 5:**
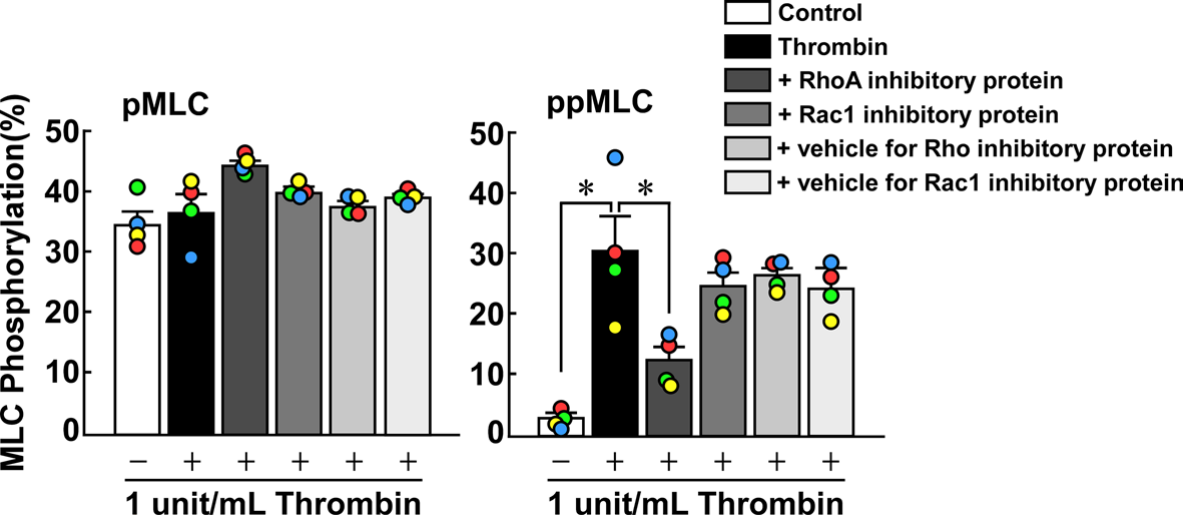
Effects of introduction of the inhibitory proteins of RhoA and Rac1/Cdc42 on the thrombin-induced mono- and di-phosphorylation of MLC (pMLC and ppMLC) in porcine aortic endothelial cells (PAECs). PAECs were pretreated with 5 μM inhibitory proteins of RhoA and Rac1/Cdc42 for 30 min prior to and during stimulation with 1 unit/mL thrombin. MLC phosphorylation was evaluated with Phos-tag SDS-PAGE followed by immunoblot detection of MLC. The data are expressed as the mean ± SEM (*n* = 4). *P < 0.05, according to an ANOVA followed by Dunnett’s post hoc test. All original images of the immunoblot analyses are shown in Additional file 1: Figure S5

The involvement of RhoA in the thrombin-induced MLC phosphorylation was directly evaluated by introducing a RhoA-inhibitory protein with help of the cell-penetrating peptide [18, 19]. The incubation of the cells with the inhibitory protein of RhoA, but not Rac1/Cdc42, 30 min prior and during 3-min thrombin stimulation inhibited the di-, but not mono-phosphorylation of MLC (Fig. 5). The inhibitory proteins of RhoA and Rac1/Cdc42 used in the present study have previously been shown to bind to their respective GTP-bound forms in the pull-down assay [18, 19].

### Knockdown of either RhoA, RhoB or RhoC alone failed to inhibit thrombin-induced di-phosphorylation of MLC in PAECs

**Fig. 6:**
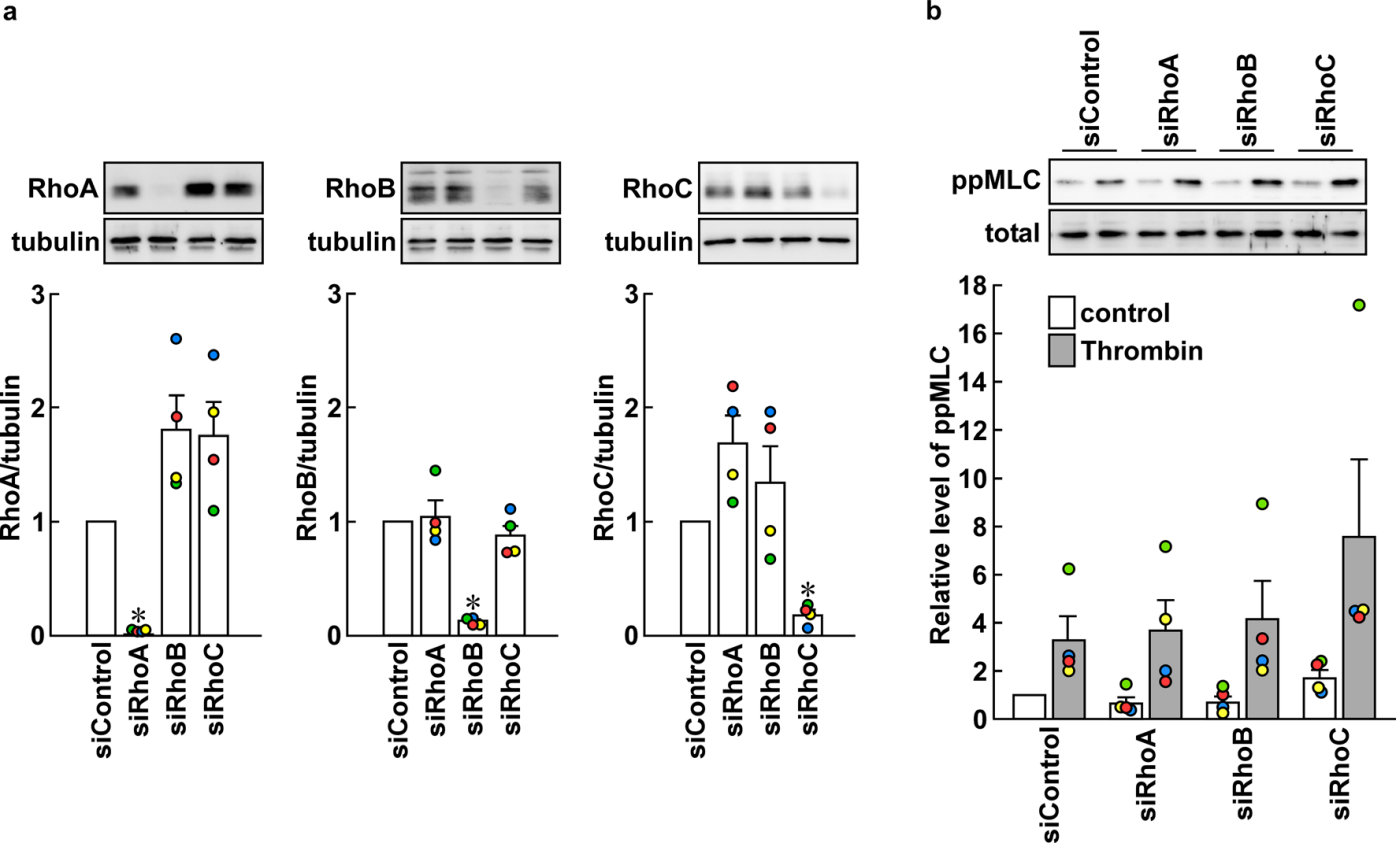
Effect of knockdown of RhoA, RhoB and RhoC on thrombin-induced di-phosphorylation of myosin light chain (ppMMLC) in porcine aortic endothelial cells (PAECs). Representative immunoblot images and summaries of immunoblot detection of RhoA, RhoB and RhoC (**a**), and ppMLC before and 3 min after stimulation with 1 unit/mL thrombin (**b**). The samples obtained before thrombin stimulation were used to detect Rho proteins. The data are expressed as mean ± SEM (*n* = 4). *P < 0.05 vs. the other conditions according to an ANOVA followed by Fisher post hoc test. In b, the values of siRhoA, siRhoB and siRhoC before and after thrombin stimulation showed no significant difference from those of siControl, according to an ANOVA followed by Dunnett’s post hoc text. All original images of the immunoblot analyses are shown in Additional file 1: Figure S6

In order to examine the specific involvement of RhoA, the effect of knockdown of RhoA, RhoB and RhoC on MLC di-phosphorylation was examined. The specific knockdown of the siRNA-targeted protein was confirmed (Fig. 6a). Knockdown of either one of RhoA, RhoB or RhoC had no effect on the levels of MLC di-phosphorylation seen before and 3 min after stimulation with 1 unit/mL thrombin (Fig. 6b). The findings suggest redundant role of three Rho proteins, but not specific involvement of RhoA.

## Discussion

The present study showed that simvastatin, an inhibitor of HMG-CoA reductase, prevented the MLC di-phosphorylation and actin bundle formation at the cell periphery in the early phase of thrombin-induced barrier disruption and also protected the thrombin-induced endothelial barrier function. The preventive effects of simvastatin were cancelled by co-treatment with GGPP, but not FPP. The protein geranylgeranylation is essential for membrane localization and the signaling function of RhoA. The present study also demonstrated that thrombin simulation indeed activated RhoA and ROCK and that the introduction of RhoA-inhibitory protein inhibited the thrombin-induced di-phosphorylation of MLC. However, knockdown of either one of RhoA, RhoB or RhoC failed to inhibit MLC di-phosphorylation. Collectively, the findings of the present study suggest that the redundant contribution of RhoA, RhoB and RhoC, but not specific involvement of RhoA, as an up-stream factor for ROCK activation. The role of Rho protein–ROCK has been well established in stress fiber formation [22]. The novelty of the present study thus resides in our clarification of the role of Rho proteins in the early events characterized by MLC di-phosphorylation and actin bundle formation at the cell periphery.

Many kinases have been shown to phosphorylate MLC in the cellular context [8, 9]. Among them, MLCK, ROCK, ZIPK and ILK have been shown to induce di-phosphorylation [8, 9]. In our previous study, the transient elevation of MLC di-phosphorylation in the early phase was wholly sensitive to inhibitors of ROCK, while MLC mono-phosphorylation was resistant in the early phase but partly sensitive in the late phase [7]. The present study showed that thrombin does indeed activate ROCK in the early phase, as evidenced by an increase in the phosphorylation of MYPT1 at the site specific to ROCK. ROCK contributes to MLC phosphorylation either by directly phosphorylating MLC or by phosphorylating MYPT1 or CPI-17, thereby inhibiting MLC phosphatase activity [8, 9]. ROCK inhibition of MLC phosphatase assists in the increased phosphorylation of MLC, thereby contributing to di-phosphorylation. The findings of our present and previous studies [7] suggest that the mono- and di-phosphorylation of MLC are independently regulated, as they showed distinct subcellular localizations, temporal profiles and sensitivities to ROCK inhibition and simvastatin. While it is difficult to clearly discriminate the direct and indirect contribution of ROCK to MLC di-phosphorylation, the direct phosphorylating effect of ROCK appears to play a major role in the MLC di-phosphorylation in the early phase.

The activity of ROCK is primarily regulated by the binding of the GTP-bound form of RhoA as well as RhoB and RhoC, which release the N-terminal catalytic domain from auto-inhibition mediated by the PH domain in the C-terminal region [13, 23]. The Rho protein-independent mechanism of ROCK activation has also been clarified. The proteolytic removal of the PH domain by caspase-3 in ROCK1 and by granzyme B in ROCK2 releases the constitutively active catalytic domain [13, 23]. Furthermore, some lipids, including arachidonic acid, presumably bind to the PH domain and activate ROCK [14, 15]. The present study demonstrated that the inhibitory protein of RhoA, which was derived from the RhoA-binding domain of ROCK [18–21], inhibited the thrombin-induced di-phosphorylation of MLC. As the RhoA-binding domain of ROCK is shared by RhoB and RhoC, the inhibitory peptide is considered to inhibit the activation of ROCK not only by RhoA, but also by RhoB and RhoC [24]. In fact, knockdown of either one of RhoA, RhoB or RhoC failed to inhibit MLC di-phosphorylation. Although specific contribution of any one of Rho proteins remains elusive, it is suggested at least that Rho proteins, but not other mechanisms, play a major role in the activation of ROCK in the early phase of thrombin-induced barrier disruption.

The mechanism that restricts MLC di-phosphorylation at the cell periphery during the early phase of thrombin-induced barrier disruption remains to be elucidated. One or some of the molecules involved in the signaling pathway from thrombin receptor PAR_1_ through Rho-GEFs and Rho proteins to ROCK are suspected to play a role. The activation of Rho-GEFs is believed to take place at the plasma membrane, as this process depends on the interaction with G protein, which is activated by the receptor [25–27]. However, their localization at the membrane is not considered to be obligatory but secondary to the interaction with G protein [25–27]. In contrast, the membrane localization appears to be obligatory for Rho proteins, especially for its GTP-bound form, as this molecule has a structure that directs the membrane localization [25–27]. In this case, geranylgeranylation plays a critical role. Statin treatment not only inhibits the cholesterol synthesis, but also depletes the intermediate metabolites, including FPP, which is further metabolized to generate GGPP, thereby inhibiting protein prenylation [27, 28]. The disappearance of MLC di-phosphorylation at the cell periphery after statin treatment and its recovery by supplementation of GGPP observed in the present study are consistent with this process. The geranylgeranylation of Rho proteins is also required for the interaction with its binding partners, including Rho GDP dissociation inhibitor and downstream effector molecules [27, 28]. Therefore, statin treatment has been reported to be associated with an increase in the GTP-bound form of Rho proteins, which is non-functional in terms of cell signaling despite its GTP-bound state [28, 29]. The expected loss of membrane localization as well as functional defect of Rho proteins after simvastatin treatment are suggested to contribute to the loss of MLC di-phosphorylation at the cell periphery in the early phase after thrombin stimulation. The absence of ectopic MLC di-phosphorylation, which might occur in the cytosol in a compensatory manner due to the loss of peripheral MLC di-phosphorylation, supports not only the localization, but also the functional defect of Rho proteins after simvastatin treatment.

Endothelial barrier function plays an important role in regulating vascular permeability essentially throughout the vasculature. The disruption of barrier function of microvascular endothelial cells plays a particular role in the pathological situations such as inflammation. Since most of the results of the present study were obtained using PAECs, we examined whether the similar event takes place in microvascular endothelial cells by using HMVECs. The actin bundle formation at cell periphery was observed as an early event in HMVECs as in the case of PAECs. Therefore, the observations obtained with PAECs may be applicable to other vasculature, at least in microvasculature. However, the peripheral actin bundle formation was observed 15 min after thrombin stimulation in the case of HMVECs. The reason for why HMVECs took longer time to respond to thrombin remains unsolved.

The membrane localization of ROCK might be secondary to that of the active form of Rho proteins [26]. However, ROCK contains a PH domain in its structure, which serves as a lipid binding domain [13, 30]. The intrinsic membrane localization of ROCK might primarily contribute to the localization of MLC di-phosphorylation at the cell periphery. The loss of MLC di-phosphorylation at the cell periphery after statin treatment is suggested to be primarily due to the loss of membrane localization of Rho proteins. However, the primary role of membrane localization of ROCK in membrane-restricted MLC di-phosphorylation remains to be determined.

The reorganization of peripheral actin bundles seen in the early phase to stress fibers in the later phase is intriguing. However, the underlying mechanism remains elusive. The organization of actin cytoskeleton occurs as a consequence of concerted action of nucleation, elongation and cross-linking or bundling in a spatio-temporary specific manner [31–34]. Rho GTPase family, including Rho, Rac and Cdc42, are thought to play a critical role in these processes [31–34]. According to the observations of the present and previous studies [7], Rho proteins appear to play a critical role in the formation of both peripheral actin bundles and stress fibers. The actin nucleation mediated by an Arp2/3 complex plays a crucial role in the formation of actin networks at adherence junctions, and the nucleation mediated by formins, such as mDia, plays an important role in the formation of linear actin bundles lining underneath [31–34]. The former is more relevant to the initial generation of adherence junctions by mediating lamellipodial activity, while the latter is more relevant to cortical actin filaments formation that characterize the actin cytoskeleton in the quiescent endothelial cells at confluence [32, 33]. The formation of peripheral actin bundles in the early phase is considered to be a continuous expansion of the cortical actin filaments. The nucleation and elongation of actin filaments by mDia are speculated to contribute to formation of the peripheral actin bundles, and myosin contributes to bundling and force generation during the early phase of barrier disruption [31, 32, 34]. In contrast, the stress fibers are composed of linear actin filaments with non-uniform polarity [31, 32]. There is little evidence for prominent contribution of Arp2/3 complex to the nucleation of stress fibers [31]. Formins, such as mDia, are implicated in nucleation and elongation of stress fibers [31, 32]. Myosin also contributes to bundling and force generation in the stress fibers [32, 33]. The putative contribution of mDia and myosin to both filaments is consistent with the Rho proteins and ROCK-dependent regulation of the activity of mDia and myosin [22]. It is therefore plausible that the spatio-temporal regulation of these factors specifies transition from peripheral actin bundles to stress fibers. However, precise mechanism by which the transition takes place remains to be elucidated.

## Conclusions

Thrombin induces MLC di-phosphorylation and actin filament formation at the cell periphery in the early phase of barrier disruption. The Rho proteins–ROCK pathway is well known to contribute to stress fiber formation, which is observed during thrombin-induced barrier disruption, especially in the late phase [7]. Our present and previous findings clarified the role of Rho proteins and ROCK in the MLC di-phosphorylation and actin bundle formation during the early phase. The membrane localization of Rho proteins due to a geranylgeranyl moiety is suggested to be a primary determinant of MLC di-phosphorylation at the cell periphery.

### Supplementary Information

**Additional file 1: Figure S1.** Original images of western blot for Figure 1c. The colors correspond to those used for data points in Figure 1c. The images with double circle was used as a representative image in Figure 1c. **Figure S2.** Original images of western blot for Figures 2a and 2b. The colors correspond to those used for data points in Figure 2. **Figure S3.** Representative images from additional 2 sets of experiments in Figure 3. The fluorescence intensities of the extracted images of ppMLC and actin, shown in gray scale, are indicated at lower right corner of each image. **Figure S4.** Original images of western blot for Figures 4a, 4b and 4c. The colors correspond to those used for data points in Figure 4. The images with double circle was used as a representative image in Figures 4a, 4b and 4c, respectively. **Figure S5.** Original images of western blot for Figure 5. The colors correspond to those used for data points in Figure 5. **Figure S6.** Original images of western blot for Figure 6. Panels a and b correspond to panel a and b of Figure 6, respectively. The colors correspond to those used for data points in Figure 6. The images with double circle was used as a representative image in Figure 6. **Figure S7.** Thrombin-induced peripheral actin bundle formation in human microvascular endothelial cells (HMVECs). Images of confocal microphotographs obtained before (Control) and 15 min after stimulation with 1 unit/mL thrombin in three independent experiments (a, b, and c) show the staining of F-actin, VE-cadherin and nuclei (TO-PRO-3) in the indicated colors.

## Data Availability

All data generated or analyzed during this study are included in this published article and its supplementary information files. The raw data supporting the conclusions of this manuscript will be made available by the authors, without undue reservation, to any qualified researcher.

## Abbreviations

ANOVA: Analysis of variance
FPP: Farnesyl pyrophosphate
GGPP: Geranylgeranyl pyrophosphate
HBS: Hepes-buffered saline
MLC: Myosin light chain
MLCK: Ca^2+^-calmodulin-dependent MLC kinase
MYPT1: Myosin phosphatase target subunit 1
PAECs: Porcine aortic endothelial cells
p-APMSF: 4-Amidinophenylmethanesulfonyl fluoride
PBS: Phosphate-buffered saline
PH: Pleckstrin-homology
pMLC: Mono-phosphorylation of myosin light chain
ppMLC: Di-phosphorylation of myosin light chain
ROCK: Rho-associated coiled-coil containing protein kinase
SDS-PAGE: Sodium dodecyl sulfate–polyacrylamide gel electrophoresis
TBS: Tris-buffered saline
TEER: Trans-endothelial electrical resistance
T-PBS: Phosphate-buffered saline containing 0.1% Tween-20
T-TBS: Tris-buffered saline containing 0.05% Tween 20
ZIPK: Zipper-interacting protein kinase
