# Supplementary material for: Critical role of Rho proteins in myosin light chain di-phosphorylation during early phase of endothelial barrier disruption
Source: J Physiol Sci. 2022 Dec 7;72:32. doi: 10.1186/s12576-022-00857-x (PMC10717653; doi:10.1186/s12576-022-00857-x)

Supplemental information for

Critical Role of Rho proteins in Myosin Light Chain Di-phosphorylation during Early Phase of Endothelial Barrier Disruption

by Mayumi Hirano and Katsuya Hirano

Department of Cardiovascular Physiology, Faculty of Medicine, Kagawa University, Miki-cho, Kita-gun, Kagawa, Japan; Hirano.katsuya@kagawa-u.ac.jp

Supplemental Figure S1. Original images of western blot for Figure 1c.
The colors correspond to those used for data points in Figure 1c. The images with double circle was used as a representative image in Figure 1c.

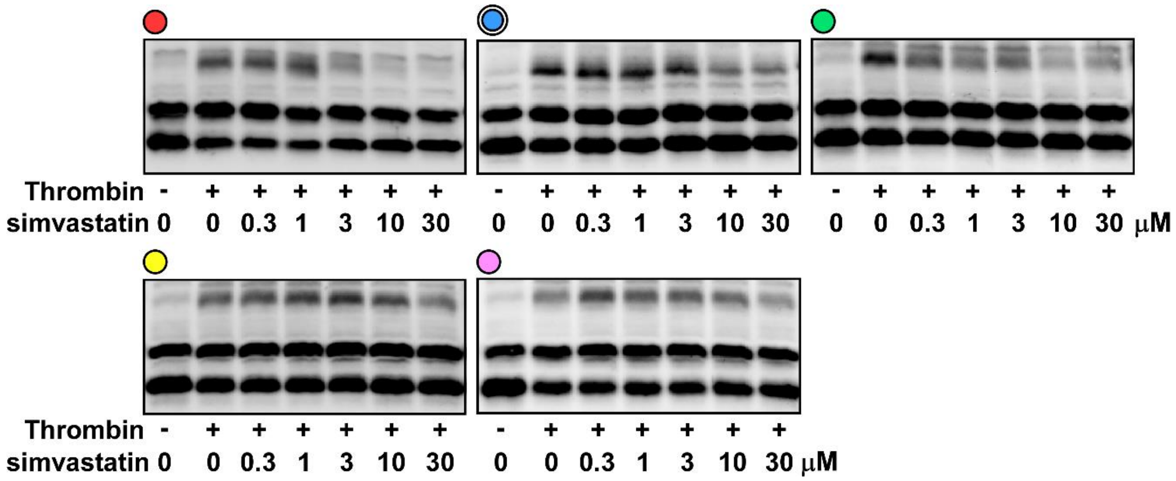

Supplemental Figure S2. Original images of western blot for Figures 2a and 2b. The colors correspond to those used for data points in Figure 2.

Original images of western blot for Fig.2a

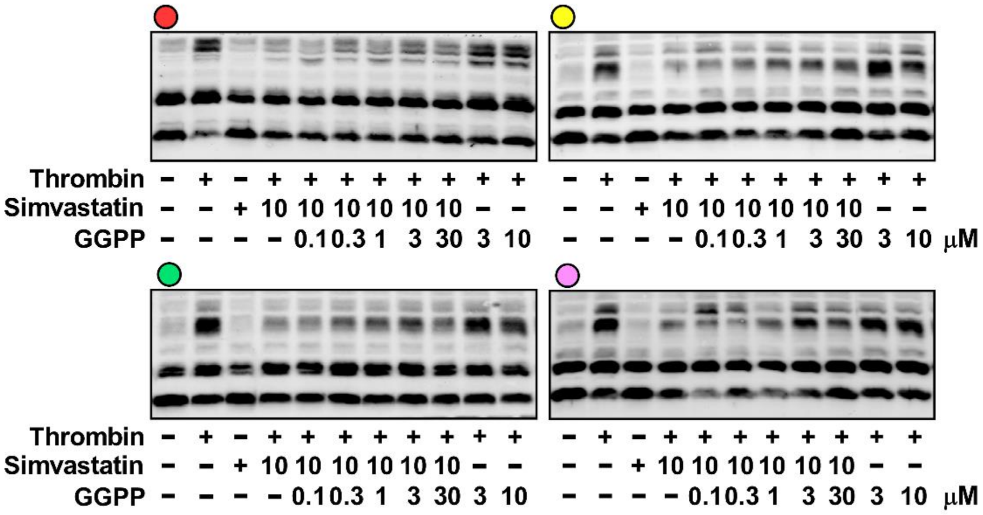

Original images of western blot for Fig.2b

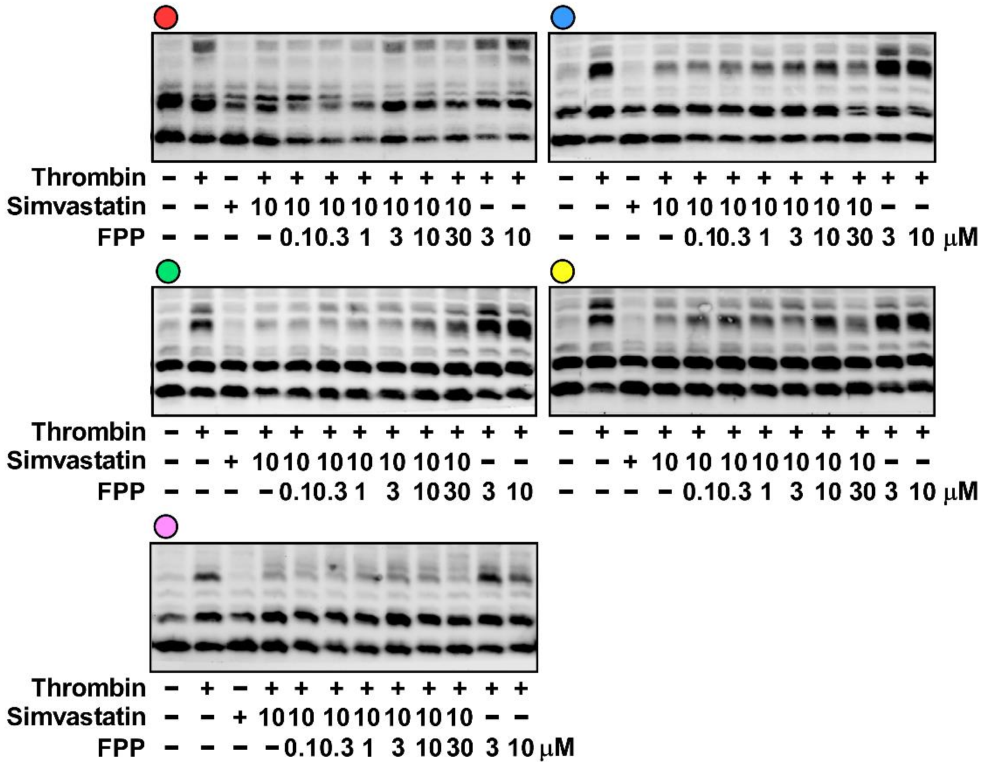

Supplemental Figure S3. Representative images from additional 2 sets of experiments in Figure 3. The fluorescence intensities of the extracted images of ppMLC and actin, shown in gray scale, are indicated at lower right corner of each image.

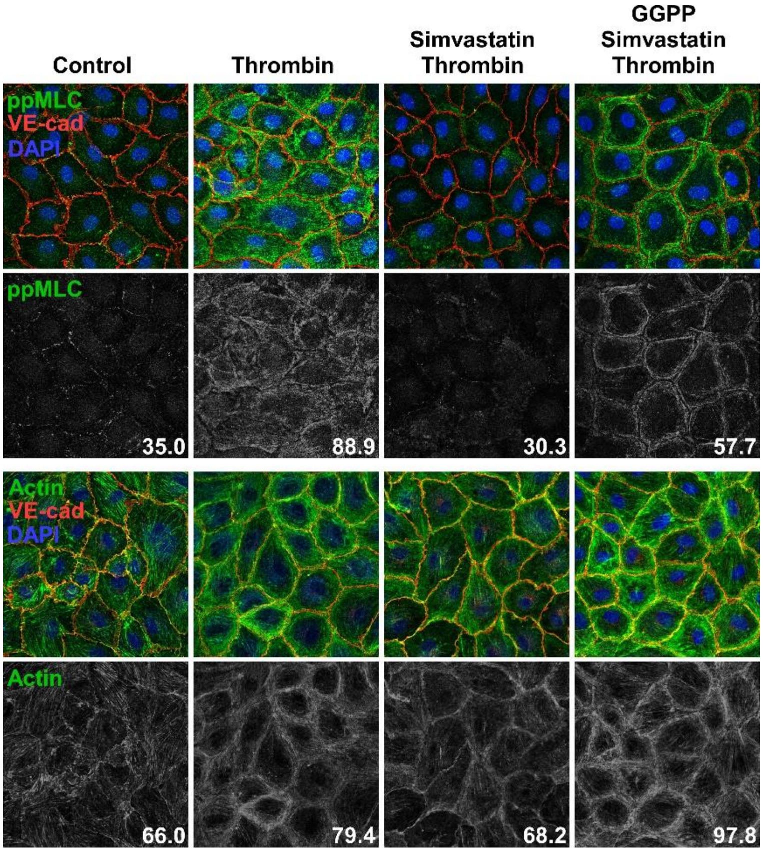

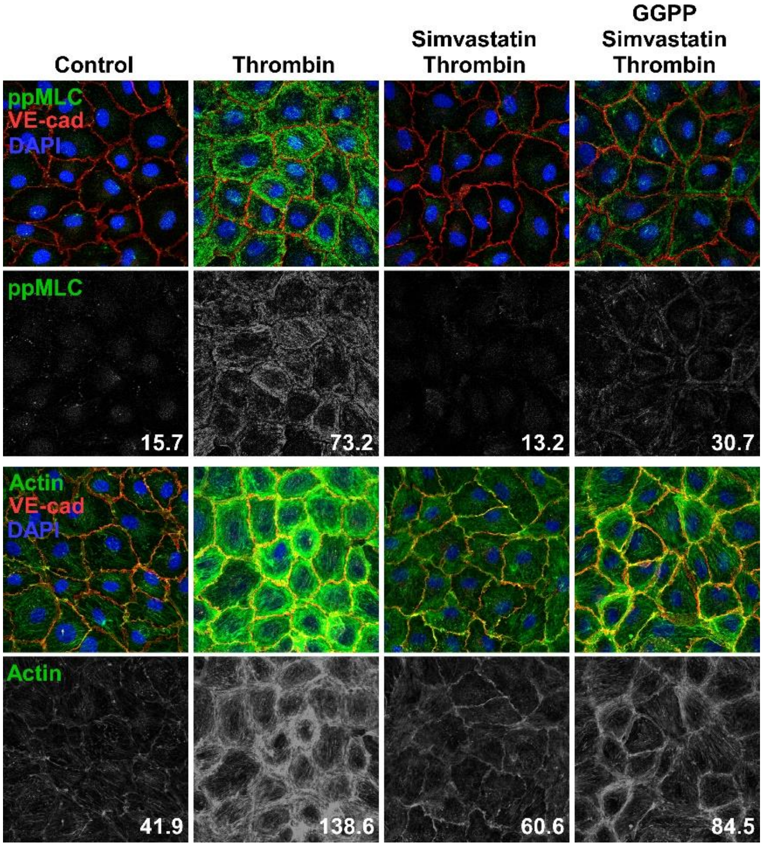

Supplemental Figure S4. Original images of western blot for Figures 4a, 4b and 4c. The colors correspond to those used for data points in Figure 4. The images with double circle was used as a representative image in Figures 4a, 4b and 4c, respectively.

Original images of western blot for Fig. 4a

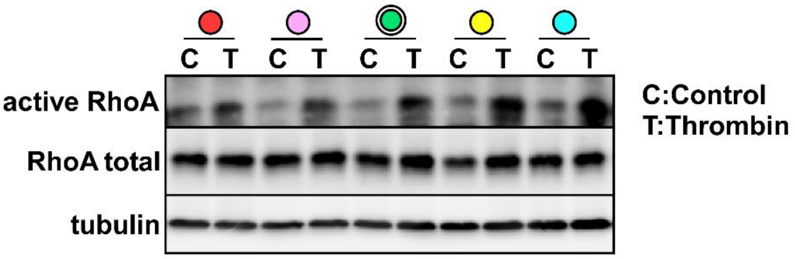

Original images of western blot for Fig. 4b

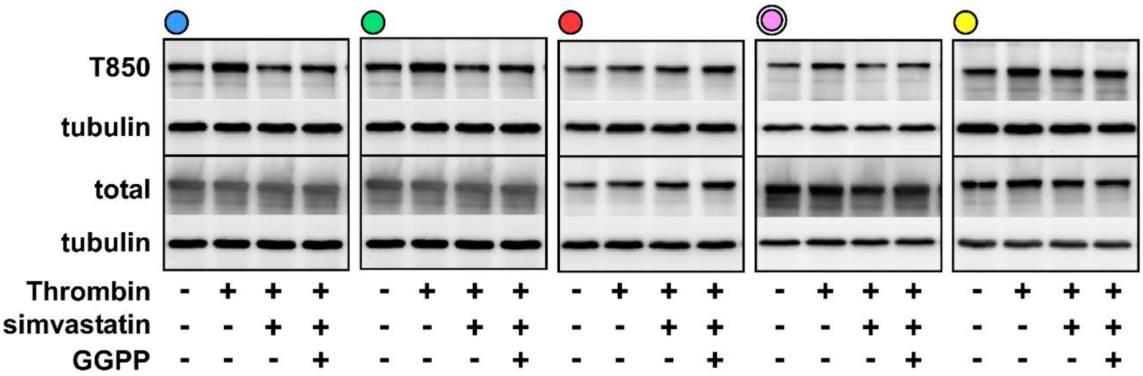

Original images of western blot for Fig. 4c

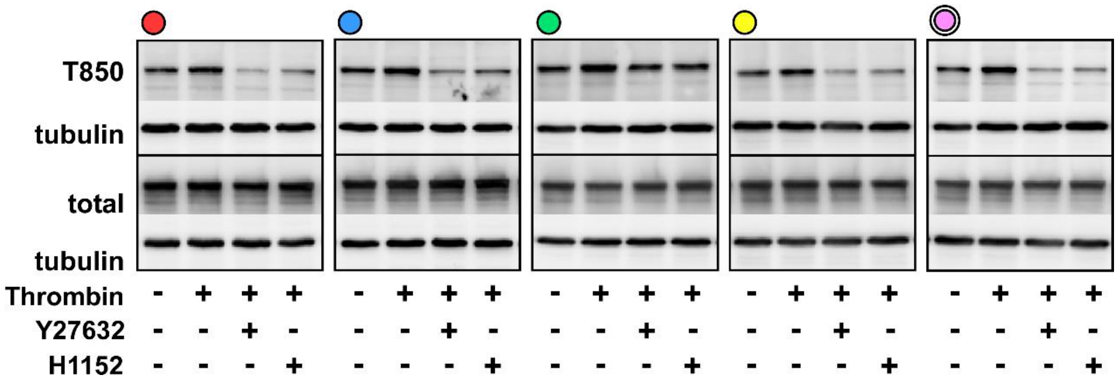

Supplemental Figure S5. Original images of western blot for Figure 5. The colors correspond to those used for data points in Figure 5.

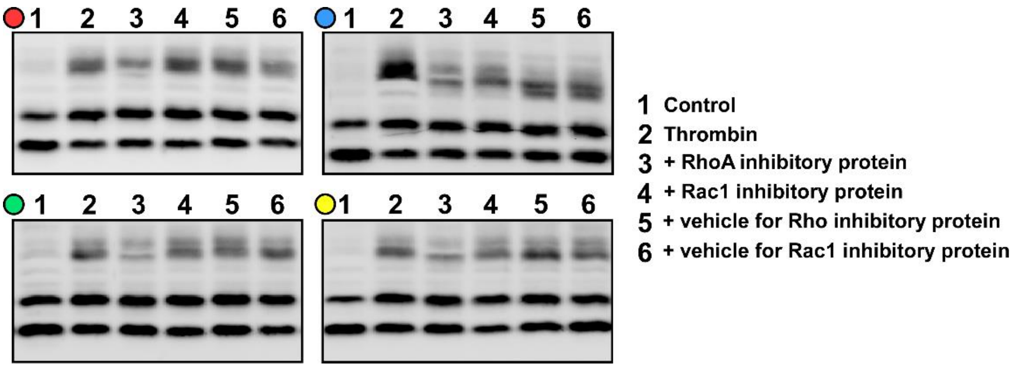

Supplemental Figure S6. Original images of western blot for Figure 6.

Panels a and b correspond to panel a and b of Figure 6, respectively. The colors correspond to those used for data points in Figure 6. The images with double circle was used as a representative image in Figure 6.

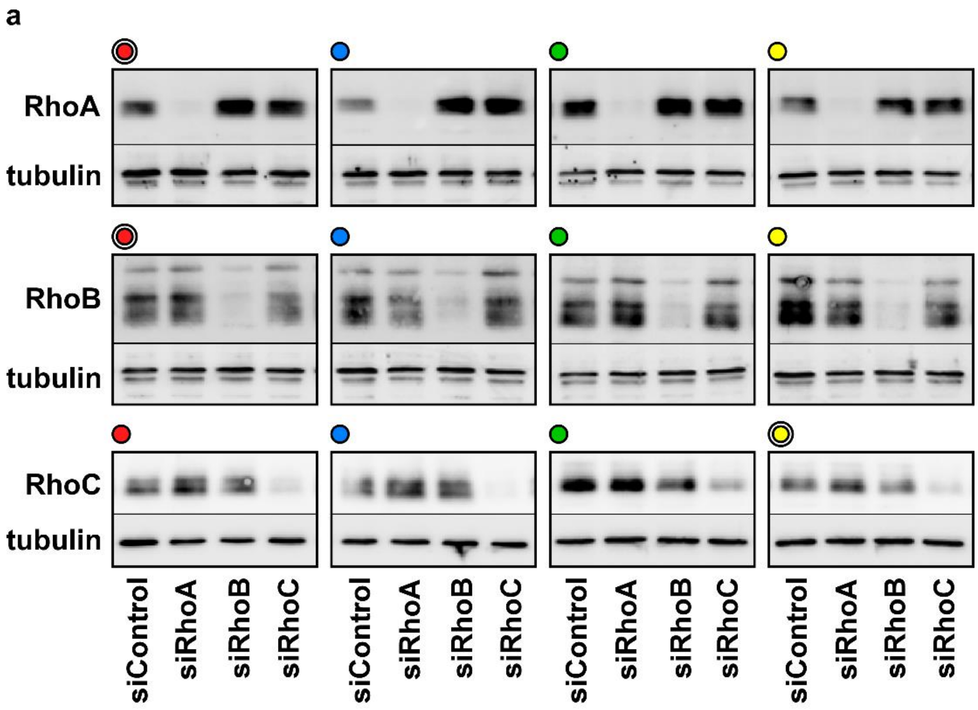

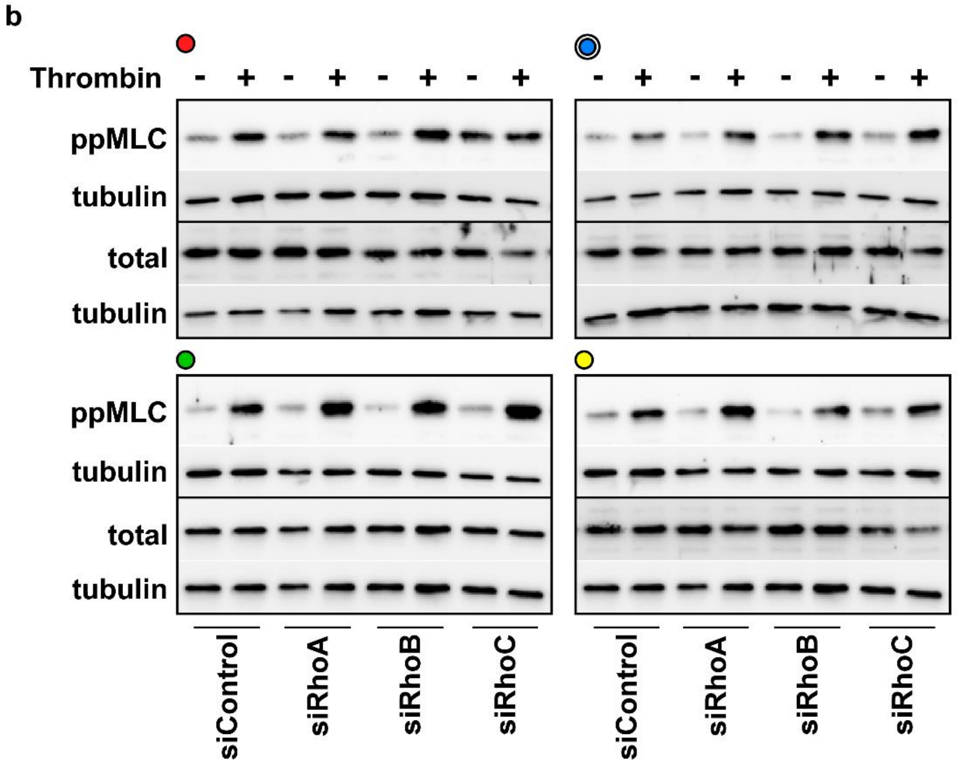

Supplemental Figure VII. Thrombin-induced peripheral actin bundle formation in human microvascular endothelial cells (HMVECs).

Images of confocal microphotographs obtained before (Control) and 15 min after stimulation with 1 unit/mL thrombin in three independent experiments (a, b, and c) show the staining of F-actin, VE-cadherin and nuclei (TO-PRO-3) in the indicated colors.

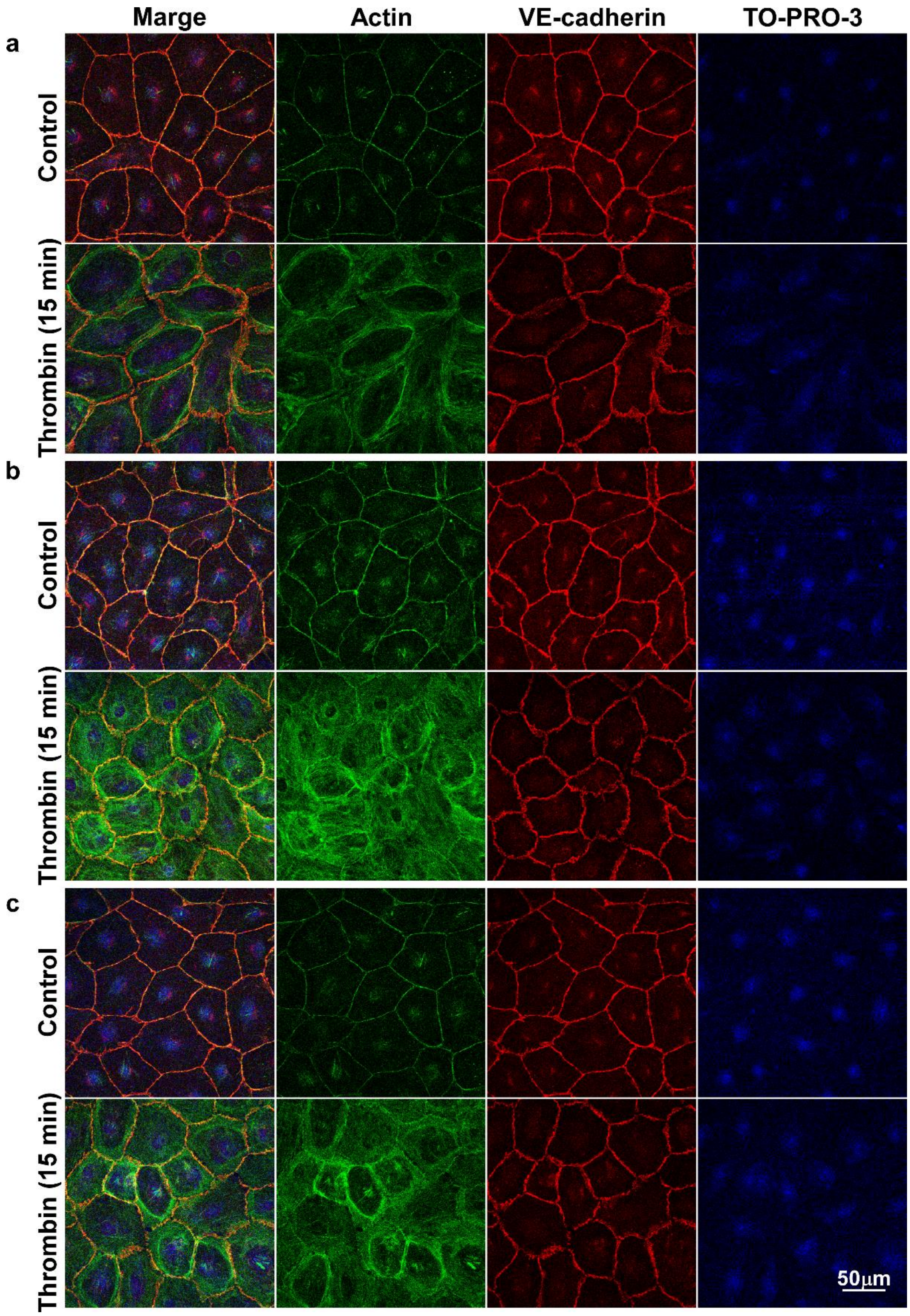

Supplement: Supplementary file 1 — Additional file 1: Figure S1. Original images of western blot for Figure 1c. The colors correspond to those used for data points in Figure 1c. The images with double circle was used as a representative image in Figure 1c. Figure S2. Original images of western blot for Figures 2a and 2b. The colors correspond to those used for data points in Figure 2. Figure S3. Representative images from additional 2 sets of experiments in Figure 3. The fluorescence intensities of the extracted images of ppMLC and actin, shown in gray scale, are indicated at lower right corner of each image. Figure S4. Original images of western blot for Figures 4a, 4b and 4c. The colors correspond to those used for data points in Figure 4. The images with double circle was used as a representative image in Figures 4a, 4b and 4c, respectively. Figure S5. Original images of western blot for Figure 5. The colors correspond to those used for data points in Figure 5. Figure S6. Original images of western blot for Figure 6. Panels a and b correspond to panel a and b of Figure 6, respectively. The colors correspond to those used for data points in Figure 6. The images with double circle was used as a representative image in Figure 6. Figure S7. Thrombin-induced peripheral actin bundle formation in human microvascular endothelial cells (HMVECs). Images of confocal microphotographs obtained before (Control) and 15 min after stimulation with 1 unit/mL thrombin in three independent experiments (a, b, and c) show the staining of F-actin, VE-cadherin and nuclei (TO-PRO-3) in the indicated colors. [file 12576_2022_857_MOESM1_ESM.pdf]
